# Identification and Characterization of Differentially Expressed Transcripts in the Gills of Freshwater Prawn (*Macrobrachium rosenbergii*) under Salt Stress

**DOI:** 10.1100/2012/149361

**Published:** 2012-04-19

**Authors:** Hirak Kumar Barman, Swagat Kumar Patra, Varsha Das, Shibani Dutta Mohapatra, Pallipuram Jayasankar, Chinmayee Mohapatra, Ramya Mohanta, Rudra Prasanna Panda, Surya Narayan Rath

**Affiliations:** ^1^Fish Genetics and Biotechnology Division, Central Institute of Freshwater Aquaculture (Indian Council of Agricultural Research), Kausalyaganga, Bhubaneswar, Odisha 751002, India; ^2^Department of Bioinformatics, Centre for Post Graduate Studies, Orissa University of Agriculture & Technology, Bhubaneswar, Odisha 751003, India

## Abstract

The giant freshwater prawn, *Macrobrachium rosenbergii,* is an economically important species. It is a euryhaline shrimp, surviving in wide-range salinity conditions. A change in gene expression has been suggested as an important component for stress management. To better understand the osmoregulatory mechanisms mediated by the gill, a subtractive and suppressive hybridization (SSH) tool was used to identify expressed transcripts linked to adaptations in saline water. A total of 117 transcripts represented potentially expressed under salinity conditions. BLAST analysis identified 22% as known genes, 9% as uncharacterized showing homologous to unannotated ESTs, and 69% as unknown sequences. All the identified known genes representing broad spectrum of biological pathways were particularly linked to stress tolerance including salinity tolerance. Expression analysis of 10 known genes and 7 unknown/uncharacterized genes suggested their upregulation in the gills of prawn exposed to saline water as compared to control indicating that these are likely to be associated with salinity acclimation. Rapid amplification of cDNA ends (RACE) was used for obtaining full-length cDNA of MRSW-40 clone that was highly upregulated during salt exposure. The sequenced ESTs presented here will have potential implications for future understanding about salinity acclimation and/or tolerance of the prawn.

## 1. Introduction

The giant river prawn, *Macrobrachium rosenbergii,* is a commercially important shrimp widely distributed in India and neighboring countries. Euryhalinity is the ability of an aquatic organism to tolerate wide salinity variations without compromising life process. *M. rosenbergii *is a euryhaline species that can survive in a wide range of salinity conditions. *M. rosenbergii* grows in freshwater but migrates to saline water (*∼*10 ppt) for the purposes of breeding and subsequent nursery rearing of larvae [1]. The estuarine environment is essential for the completion of larval metamorphosis and thereafter postlarvae undertake a return migration to the riverine region [2]. Migration is believed to be associated with the process of adaptability in different saline conditions. This species experiences different local microenvironments, especially salinity and temperature, during migration. As a result, they must have developed an osmoregulatory adaptive mechanism so as to survive in different environmental (saline) conditions.

Since *M. rosenbergii* migrates between brackish and fresh waters during their life cycle, this species will be suitable for studying underlining biochemical, physiological, and gene regulatory pathways. A change in gene expression has been an important component for stress management of living organisms [3–6]. However, very little information is available about variations in gene expressions related to salinity tolerance in *M. rosenbergii*.

Gill plays a significant role in modulating ion transport either during freshwater to saline water acclimation or vice versa [6–9]. A substantial decrease in K-phosphatase activity including lowered *α*-subunit protein levels of Na, K-ATPase, and a redistribution of enzyme activity into membrane fractions of different densities in salinity-acclimated shrimp (*M. amazonicum*) was documented [10]. Thus, gill tissues from shrimp exposed to fresh and saltwater should be one of the ideal organs for comparative genetic analysis of the molecular basis of physiological acclimatization. Most of the studies were restricted to one or small number of genes. The mechanisms of adaptability to saline water are expected to be a complex physiological process associated with branchial ion transport. It is essential to understand the complex physiological roles played by the gill.

The analysis of differential gene expression in the gill of shrimp exposed to saline water can reveal adaptive mechanisms to salinity stress. Identification of salinity-induced expression of transcripts in the gill will provide information about gill-specific adaptive response of the shrimp. Subtractive and suppressive hybridization (SSH) technique is an effective approach in identifying differentially expressed stress-induced genes in plants, fishes, and crustaceans [11–15], especially when prior sequence knowledge is lacking.

In the absence of prior sequence knowledge for *M. rosenbergii,* a salinity-enriched cDNA library was generated from the gill, by using SSH technique, in order to identify salinity-tolerant transcripts. It was possible to generate sequence information of several ESTs; those are likely to be linked with regulatory pathways during salt water acclimatization. A subset of forward subtracted transcripts were upregulated as examined by quantitative real-time PCR (qPCR) for differential expression in gill exposed to saltwater by comparing with those cultured in fresh water. The full-length cDNA sequence information was generated for one of those transcripts being highly upregulated.

## 2. Materials and Methods

### 2.1. Salt Treatment

Adult *M. rosenbergii *were exposed to either salt water at 10 ppt and 24 ppt levels (freshwater was adjusted with sea water) or fresh water (0 ppt salinity) for 24 days in fiberglass-reinforced plastics (FRP) tank (3 separate tanks for 0 ppt, 10 ppt, and 24 ppt with 10 individuals in each tank) commonly being used for breeding purpose. After 24 days of treatments, gills of each individual were frozen in liquid nitrogen for total RNA extraction.

### 2.2. RNA Extraction

The mRNA extraction was performed as described earlier [16]. Briefly, frozen gill tissues of *M. rosenbergii* were ground in liquid nitrogen. Total RNA was extracted using the TRIzol reagent following manufacture's instructions (Invitrogen). DNase I-treated RNAs were precipitated and resuspended into diethyl pyrocarbonate-treated water. Equal amounts of total RNA from each saltwater or freshwater-treated condition (5 individuals of each group) were mixed. The quantity and quality of isolated total RNA were examined by spectrophotometric readings and denaturing formamide/agarose gel electrophoresis. Poly-A tailed RNA enrichment was carried out using mRNA purification kit (Sigma, Aldrich, USA).

### 2.3. Construction of Subtracted cDNA Library

SSH was performed using PCR-select subtraction kit (Clontech, USA) as described [17] with slight modifications. Briefly, cDNA was reversely transcribed from 1.5 *μ*g of the mixed mRNA mentioned above using Mint cDNA synthesis Kit (Evrogen). Saltwater-treated cDNA was considered as tester and freshwater was treated as driver. Tester and driver cDNA were digested with *Rsa*I, followed by adapter ligation to tester cDNA. Following two rounds of hybridization, primary and nested PCR amplifications were carried out for 27 and 12 cycles, respectively, to normalize and enrich the differentially expressed cDNAs. Subtractive efficiency was checked by PCR amplification (18, 23, 28, 33, and 38 cycles) of the *β*-actin gene on subtracted and unsubtracted cDNA. The forward-subtracted secondary PCR products were purified and ligated into pGEM-T easy vector (Promega, USA) and transformed into *Escherichia coli *DH5*α* competent cells and plated onto agar plates to generate a subtracted cDNA library.

### 2.4. Quantitative Real-Time PCR (qPCR)

Quantitative real-time PCR (qPCR) for ESTs and the house-keeping genes were performed in triplicate for each cDNA sample using Light Cycler-480 SYBR Green I kit (Roche Diagnostics, Germany) in a Light Cycler 480 RT-PCR instrument (Roche Diagnostics, Germany) as described in [16, 18]. The qPCR was performed on cDNA generated from RNA obtained from the pooled RNA samples. Relative mRNA levels of target genes were normalized to RpL8 expression for each sample, and normalized standard deviations were calculated. Primer annealing temperature for target genes and RpL8 was 58°C. Negative control PCR containing RNA template and RpL8 primers was performed for each sample to rule out the possibility of genomic DNA contamination.

### 2.5. Rapid Amplification of cDNA Ends (RACEs)

Since the expressions of MRSW-40 transcript were dramatically elevated to the tune of ~15-fold in saline-exposed prawn as compared to control (0 ppt salinity) fishes, its full-length cDNA sequence information was generated, by 5′- and 3′-RACE. RACE-PCR was performed to obtain the 5′- and 3′-ends of the MRSW-40 EST using Smarter RACE cDNA amplification kit (Clontech, USA) following protocol as described [16] with minor modifications. Gene-specific primers (GSP1 and GSP2) were designed from the obtained sequence data of MRSW-40 clone (Table 1). The first PCR was performed using GSP1 and Universal Primer A mix (UPM; provided with the kit) with program as follows: 5 cycles of 30 sec at 94°C, 3 min at 64°C (for 3′-end and for 5′-end 70°C) followed by 5 cycles of 30 sec at 94°C, 30 sec at 62°C (for 3′-end and for 5′-end 68°C), 3 min at 72°C and finally followed by 25 cycles of 30 sec at 94°C, 30 sec at 60°C (for 3′-end and for 5′-end 66°C), and 3 min at 72°C. Then, the second PCR was performed using GSP2 and Nested Universal Primer A (NUP; provided with the kit) with program as follows: 25 cycles of 30 sec at 94°C, 30 sec at 60°C (for 3′-end) and 66°C (for 5′-end), and 3 min at 72°C. PCR products were separated on a 1.5% agarose gel, and desired bands were excised for purification using gel extraction kit (USB, USA). The purified fragments were cloned into pGEMT easy vector (Promega, USA) and then transformed into chemically competent DH5*α* cells. The positive inserts were sequenced using an automated ABI 310 genetic analyzer (Perkin-Elmer Applied Bios system). The sequences were verified using the BLASTN program (http://www.ncbi.nlm.nih.gov/blast). The amino acid sequence was deduced by Expasy translate tool (http://expasy.org/tools/dna.html) and verified using BLASTP at the same site. 

## 3. Results

### 3.1. Assembly and Characterization of *M. rosenbergii* ESTs Generated by SSH

**Figure 1:**
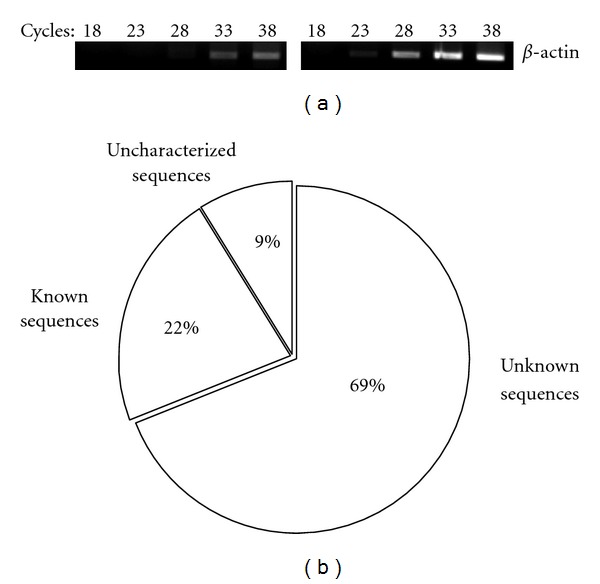
Subtraction efficiency verification and characterization of ESTs. (a) Subtraction efficiency was estimated by polymerase chain reaction (PCR) amplification of *β*-actin gene on subtracted and unsubtracted cDNA libraries. The number of PCR cycles is indicated above each lane. (b) EST classification represented in subtracted library based on sequence analysis of 117 nonredundant inserts. Known sequences exhibit significant homology with known genes. Uncharacterized sequences were homologous to unannotated EST sequences. Sequences with no significant match were called unknown sequences.

To identify the genes involved in adaptability to saline water, a forward-subtracted cDNA library was constructed from the pooled mRNA extracted from the gill of *M. rosenbergii* exposed to salt water (10 and 24 ppt) representing tester cDNA and freshwater exposure as driver. The enriched transcripts generated by SSH are expected to be the representation of salinity-induced transcripts. Subtraction efficiency was evaluated by comparing the amplified housekeeping **β*-actin* gene with the templates of subtractive and unsubtractive PCR products of second round (Figure 1(a)). In the subtracted library, 33 cycles were necessary to detect the amplified band of **β*-actin* gene compared with 23 cycles in the unsubtracted library. A substantial reduction in **β*-actin* gene cDNA to subtracted testis cDNA indicated that a large number of constitutive genes were removed effectively, and salinity-specific genes were enriched successfully.

After subtraction, the cDNA fragments were cloned, and a total of 162 randomly picked clones contained inserts, ranging from 90 bp to 850 bp, and were considered for sequencing. Of the 162 clones sequenced, 127 nonrepeated, good-quality sequences were aligned to those in the GenBank databases. Ten clones belonged to the ribosomal proteins.  Figure 1(b) shows the BLAST results for the rest of 117 gene transcripts identified and submitted to GenBank (GenBank Accession nos. JK502144–JK502260). Sixty-nine percent (81 clones) of the unique sequences exhibited no significant homology to any previously identified genes (termed unknown). About 22% (26 clones) of putative transcripts showed significant (>65%) sequence homology with vertebrate genes (termed known) as shown in Table 2, while 10 EST fragments (9%) showed homology only with unannotated ESTs (termed uncharacterized) as shown in Table 3. Most of the uncharacterized ESTs matched with crustacean ESTs generated from various organs including gill (Table 3). All the clones of known category matched with stress-induced genes including 12 clones linked to salt tolerances for fish and shellfish species (Table 2). This further validated the assertion that efficient enrichment of transcripts for salinity tolerance was achieved. Functionally known transcripts represented broad spectrum of biological pathways such as energy metabolism, signal transduction, transcription factors, and transport facilitators.

### 3.2. Relative Expression Pattern of Known and Unknown ESTs in the Gills of *M. rosenbergii* Exposed to Saline Water

To validate the outcomes of SSH-mediated enrichment of salinity-induced transcripts, qPCR was performed to quantify the abundance of selected ESTs. Differential quantifications were examined for few known and unknown transcripts in the gills of *M. rosenbergii* exposed to seawater (adjusted to 10 ppt salinity) and freshwater (0 ppt salinity). Seawater at 10 ppt salinity was chosen because hatchery-based breeding for *M. rosenbergii* in a mass scale is generally performed at around 10 ppt level. The known EST clones were selected based on earlier evidence associated with salinity-stress tolerance in other fish/crustacean and quality of sequence generated by SSH. Pooled mRNAs from the gills of ≥5 individuals from each experimental condition were selected for cDNA preparation.

Evaluation of the best reference genes for qPCR is essential. Expression stability for house-keeping genes in the larvae, postlarvae, and gill of adult *M. rosenbergii* exposed to salt water and freshwater was analyzed using geNorm rankings as described [16, 32]. In this study, the mRNA quantity levels for GAPDH, RpL8, RpL18, *β*-actin, and Elf*α* house-keeping genes were tested. Among these, expression of RpL8 was identified as the most stable (data not shown) consistent with earlier findings [6, 11], and hence, the qPCR data was normalized with RpL8.

**Figure 2:**
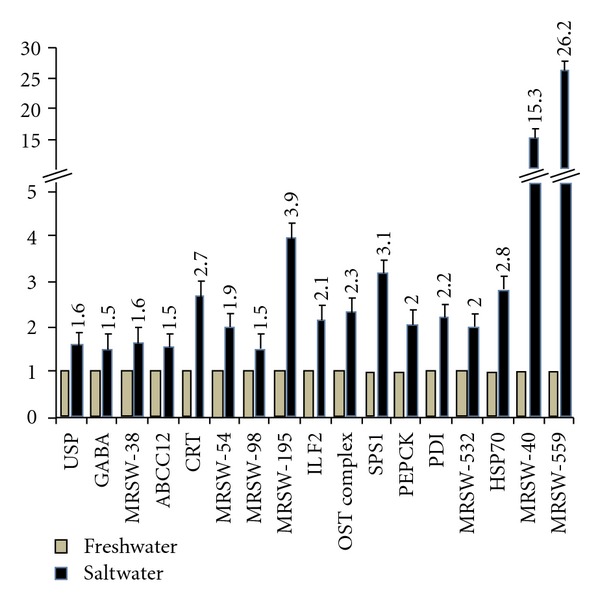
The expression profiles of selected ESTs enriched from SSH cDNA library by quantitative real-time polymerase chain reaction (qPCR). The qPCR data for all ESTs was normalized with RPL8 as reference gene. The qPCR data shows the relative gene expression levels in the salt water-stressed gill of *M. rosenbergii *over unstressed gill (default setting as 1 in each case). The clones MRSW-40 and MRSW-559 were significantly up-regulated in the tune of about 15- and 26-fold, respectively. The rest of the clones were up-regulated in the tune of ≥1.5-fold. The numbers on top of each bar for salt water-treated gill represent fold changes in expressions relative to the expressions in freshwater-treated gill. The data represents the average of three independent (each in duplicate) experiments. USP, ubiquitin-specific protease; GABA, *γ*-aminobutyric acid; ABCC12, ATP-binding cassette protein C12; CRT, calreticulin; ILF2, interleukin enhancer binding factor 2; OST complex, oligosaccharyltransferase complex; SPS1, selenophosphate synthetase 1; PEPCK, phosphoenolpyruvate carboxykinase; PDI, protein disulphide isomerase; HSP70, heat shock protein 70.

Figure 2 shows the fold change obtained between salt-exposed and control samples. As expected, the relatively elevated levels of mRNA expressions (≥1.5-fold) were detected in salt-treated gill samples more than control with 9 interested known genes such as ubiquitin-specific protease (USP, MRSW-1), protein disulfide isomerase (PDI, MRSW-350), *γ*-aminobutyric acid (GABA, MRSW-18), ATP-binding cassette protein C12 (ABCC12, MRSW-43), calreticulin (CRT, MRSW-51), interleukin enhancer binding factor 2 (ILF2, MRSW-291), oligosaccharyltransferase complex (OST complex, MRSW-293), selenophosphate synthetase 1 (SPS1, MRSW-343), and phosphoenolpyruvate carboxykinase (PEPCK, MRSW-348). Heat shock protein 70 (HSP70) was known to be upregulated in brown trout and rainbow trout while migrated to saline water [5, 33], but it possibly was not picked up as a clone in our study. HSP70 was taken as a positive control for our qPCR analysis. As expected, HSP70 expression was up-regulated in the gill of saline-treated prawns (Figure 2). These findings suggested their physiological significance with regard to osmoregulatory adaptive mechanism in diverse species including *M. rosenbergii.*

Of the unknown EST clones, the abundance of expressions for MRSW-40 and MRSW-559 clones was maximum in the tune of *∼*15- and *∼*26-fold, respectively, in salt-treated gills (Figure 2). The significantly higher level of expression for MRSW-195 was documented due to salt exposure. Salt exposure also caused increased levels (≥1.5-folds) of MRSW-38, MRSW-54, MRSW-98, and MRSW-532 mRNAs expressions in the gill. Thus, 7 unknown clones, being up-regulated in the gills of *M. rosenbergii* cultured in saline water, could be considered as novel ESTs that are most likely to be linked with salinity-stress tolerance.

### 3.3. Generation of Full-Length cDNA Sequence for MRSW-40 EST

**Figure 3:**
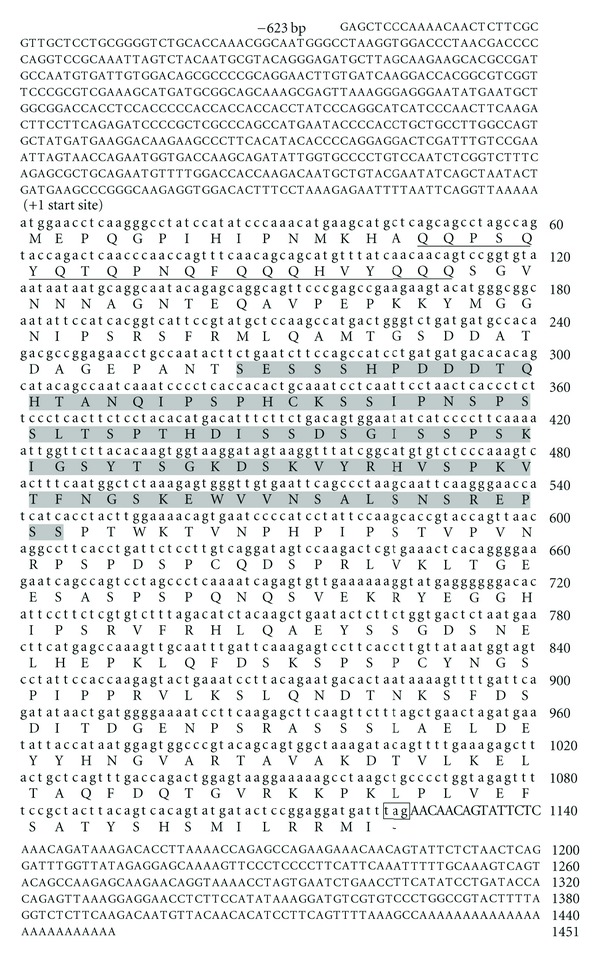
The full-length cDNA sequence of MRSW-40 EST derived through RACE and the deduced amino acid sequence. The ORF is shown in small letters which encode 374 amino acids. The in-frame stop codon in the cds is marked within a box. The 5′- and 3′-UTR are shown in capital letters. The identified glutamine-rich domain is underlined, and the serine-rich motif is shaded within gray box.

The RACE-derived full-length cDNA sequence of MRSW-40 transcript contained an open reading frame (ORF) of 1125 nucleotides translatable to 374 aa with an ATG (M) start codon and a TGA stop codon (Figure 3). The first start codon was within the consensus sequence of GTTATGG fulfilling the Kozak criteria (A/GNNATGG) [34]. The sequence consisted of 623 bp 5′-flanking region, relative to start codon, and 311 bp 3′-UTR. The poly-A tail was identified within 3′-untranslated tail. A schematic representation of deduced amino acid (aa) for clone MRSW40 is shown in Figure 3. The aa sequence derived from the clone MRSW40 was analyzed with the help of Expasy tool (www.expasy.org/tools/dna.html). Two important domains such as Glutamine- and Serine-rich corresponding to 16 to 37 aa and 89 to 182 aa, respectively, were identified. Thus, it was possible to identify and characterize full-length sequence information of one possible novel cDNA, whose expression levels are affected by salinity exposure. 

## 4. Discussion

The present study is the first attempt to explore gene expression in the gill of commercially important *M. rosenbergii* exposed to saline water. In euryhaline species, adaptability to saline water is likely to be associated with complex trait, which is controlled and regulated by many genes. Because SSH works efficiently, especially in the absence of prior sequence information, we utilized this technique for efficiently enriching a subset of genes that are up-regulated by salinity stress in the gill of *M. rosenbergii*. We have identified 26 ESTs encoding regulatory proteins associated with biological processes of salinity stress management.

The encoded phosphoenolpyruvate carboxykinase (PEPCK), the key gluconeogenic enzymes associated with energy-requiring metabolic pathway, was enriched in the cDNA library. The elevated transcriptional level of PEPCK during hyperosmotic stress was documented earlier in magur *(Clarias batrachus*) and a euryhaline crab (*Chasmagnathus granulate*) [22, 23, 35]. Protein disulfide isomerase (PDI) is also involved in metabolic pathway, and its upregulation in the gill of saline-water-exposed prawns indicated that high-energy-demanding metabolic pathways are involved in salinity acclimation. Additionally, components of signaling pathways, transporters, and transcriptional regulators such as ubiquitin-specific protease (USP), *γ*-aminobutyric acid (GABA), oligosaccharyltransferase (OST) complex, calreticulin, and elongation factor 1 were up-regulated during saline exposure demonstrating that gill is actively involved in many cellular functions for transcriptional regulation of metabolism and maintaining homeostasis for saline water adaptability. USP was reported to be involved in managing ischemic stress tolerance [19]. Here, we provide evidence that it is involved in salinity stress tolerance. Calreticulin, located in endoplasmic reticulum (ER) lumen, has been functionally linked with signal transduction and homeostasis of Ca^2+^ including acting as a protein-folding chaperone. It is involved in many biological processes of growth, reproduction, molting [36], and stress responses of temperature, oxidative, and heavy metal contamination [24]. From this study, it is evidenced that it could also be used as a biomarker for salinity stress responses. Interleukin enhancer binding factor 2 (ILF2), a known transcriptional regulator for T-cell-mediated immune defense against biotic stress caused by infection [27], is up-regulated and hence might be involved in physiological process of euryhalinity. ATP-binding cassette (ABC) transporters form one of the largest protein families. ABC containing proteins participates in regulating ion channels in the plasma membrane, especially ATP-sensitive potassium channels [21], and thus the ABC transcript identified in our study is most likely to be associated with osmoregulatory pathway. ABC protein C12 (ABCC12), a variant belonging to ABC gene family, has been cloned and characterized in mouse and human [37]. Its expression in various organs with highest in testis was documented. From this study, it appears that ABCC12 plays a significant role in salinity adaptation in the gill. However, its exact participatory role in salinity tolerance remains to elucidate. OST complex is a transporter that transfers the core oligosaccharide via *N-*linkage to an Asn residue of nascent peptide for downstream-regulated maintenance of homeostasis during salinity/osmotic stress [28]. An alternative pathway of selenocysteine (Sec) biosynthesis mediated by selenophosphate 1 (SPS1) protein exists only in aquatic arthropods including insects, but not in other eukaryotes, for oxidative stress tolerance [29]. In this study, SPS1 was found to be involved in salinity stress tolerance.

Of the 81 genes of unknown functions obtained from the SSH library, 7 selected transcripts (MRSW-38, MRSW-40, MRSW-54, MRSW-98, MRSW-195, MRSW-532, and MRSW-559) were up-regulated (≥1.5-folds) in the gill of saline-treated prawns. These transcripts are of particular interest because of their likely potential functions with salinity stress tolerances. Among these, since the expressions of MRSW-40 and MRSW-559 transcripts were dramatically elevated in the tune of more than15-fold in saline-exposed prawn as compared to control (0 ppt salinity), the full-length cDNA sequence information was generated for MRSW-40 EST to characterize and ascertain its functional relevance. The deduced 374 amino acid sequence of it contained two important domains, that is, glutamine- and serine-rich motifs. The regulatory roles of these two domains present in other proteins were associated with stress tolerance [38, 39]. It is believed that variable repeat-containing ORFs are associated with transcriptional regulation. Polymorphic proteins containing repeat sequences confer adaptive survival of the cell in the extreme environmental conditions. The presence of relatively longer 5′-UTR is most likely to be associated with regulatory noncoding regions. In this study, the knowledge gained from the full-length sequence for MRSW-40 would be of great help, in future, in dissecting regulatory role being played by it during salinity stress induction.

In conclusion, an up-regulated SSH library was constructed successfully from the gills of *M. rosenbergii* exposed to saline water. The ESTs screened from the library encode various molecules potentially associated with different biological processes, which are involved in cellular metabolic processes, signal transduction and biological regulation, response to stimuli, and other functions and unknown functions. The information generated from this study is expected to provide new insights into the salinity-mediated stress tolerance mechanisms of *M. rosenbergii*. Future studies could be undertaken to functionally validate these ESTs *in vivo* and to uncover physiological significance in response to salinity tolerance.

## Figures and Tables

**Table 1:** The gene-specific primers (GSP) designed for RACE PCR for clone MRSW-40.

RACE primers	Sequence	Size (bp)	*T*_*m*_
GSP1 (5′-RACE)	GGCGTCTGTGGCATCATCAGACCCAGTC	28	66.0°C
GSP2 (5′-RACE)	GGAGCATACGGAATGACCGTGATGG	25	61.3°C
GSP1 (3′-RACE)	CCATCCTGATGATGACACACAG	22	55.4°C
GSP2 (3′-RACE)	TCAAATCCCCTCACCACACTGC	22	59.4°C

**Table 2:** The SSH-generated cDNA clones showing significant similarity to known sequence in the public databases.

Clone number (length)	Homologue	Species	% of identity (length)	*e*-value	Function		Reference
					Stress	Regulatory action	
MRSW-1 (503 bp)	Ubiquitin-specific protease (USP)	*Pediculus humanus corporis *(body louse)	67 (257/378)	2*e* − 26	Ischemic stress tolerance	Signalling pathways	[19]

MRSW-15 (690 bp)	Protein disulphide isomerase (PDI)	*Litopenaeus vannamei *(whiteleg shrimp)	69 (117/168)	6*e* − 09	Salt tolerance	Metabolism and energy	[6]

MRSW-350 (454 bp)	Protein disulphide Isomerase (PDI)	*Scylla paramamosain *(crab)	74 (304/407)	7*e* − 69	Cold/freezing tolerance	Metabolism and energy	[6]

MRSW-18 (446 bp)	*γ*-aminobutyric acid (GABA)	*Strongylocentrotus purpuratus *(sea urchin)	66 (225/340)	2*e* − 07	Salt tolerance	Osmoregulation	[20]

MRSW-43 (934 bp)	ATP-binding cassette protein C12 (ABCC12)	*Homarus americanus *(American lobster)	75 (305/405)	2*e* − 14	Salt tolerance	Osmoregulation	[21]

MRSW-48 (179 bp)	Phosphoenolpyruvate carboxykinase (PEPCK)	*Chasmagnathus granulata *(estuarine crab)	76 (135/178)	7*e* − 33	Salt tolerance	Gluconeogenesis	[22, 23]

MRSW-348 (322 bp)	Phosphoenolpyruvate carboxykinase (PEPCK)	*Chasmagnathus granulata *(estuarine crab)	68 (208/305)	3*e* − 28	Salt tolerance	Gluconeogenesis	[22, 23]

MRSW-229 (266 bp)	Phosphoenolpyruvate carboxykinase (PEPCK)	*Chasmagnathus granulata *(estuarine crab)	70 (179/254)	2*e* − 30	Salt tolerance	Gluconeogenesis	[22, 23]

MRSW-51 (458 bp)	Calreticulin (CRT)	*Penaeus monodon *(giant tiger shrimp)	81 (370/456)	8*e* − 119	Temperature stress	Calcium signalling/homeostasis pathways	[24]

MRSW-52 (303 bp)	Betaine-homocysteine methyltransferase (BHMT)	*Danio rerio *(zebrafish)	69 (199/287)	4*e* − 19	Salt tolerance	Osmoregulation	[7]

MRSW-219 (493 bp)	Betaine homocysteine methyltransferase (BHMT)	* Homarus americanus *(American lobsters)	74 (207/278)	3*e* − 42	Salt tolerance	Osmoregulation	[7]

MRSW-58 (409 bp)	Ubiquitin (Ub)	*Portunus trituberculatus *(swimming crab)	81 (326/404)	3*e* − 105	Salt tolerance	Metabolism	[6]
MRSW-61 (366 bp)	Gastrolith protein	*Cherax quadricarinatus *(red claw fish)	73 (268/367)	2*e* − 50	Salt tolerance	Biomineralization	[25]

MRSW-68 (535 bp)	Nicotinamide adenine dinucleotide kinase (NADKs)	*Strongylocentrotus purpuratus *(sea urchin)	70 (94/134)	2*e* − 06	Cold tolerance	Energy	[26]

MRSW-92 (462 bp)	Eukaryotic translation initiation factor 3	*Danio rerio *(zebrafish)	71 (324/454)	2*e* − 51	Salt tolerance	Signalling pathway	[6]

MRSW-202 (372 bp)	Elongation factor 1-alpha	*Portunus trituberculatus *(swimming crab)	84 (311/370)	3*e* − 112	Salt tolerance	Protein synthesis	[6]

MRSW-291 (249 bp)	Interleukin enhancer binding factor 2 (ILF2)	*Eriocheir sinensis *(Chinese mitten crab)	81 (201/248)	9*e* − 07	Salt tolerance	Immune defence	[27]

MRSW-293 (493 bp)	Oligosaccharyltransferase complex (OST complex)	*Arabidopsis thaliana*	70 (268/383)	8*e* − 40	Salt and osmotic stress tolerance	*N*-linked glycosylation	[28]

MRSW-343 (262 bp)	Selenophosphate synthetase 1 (SPS1)	*Bombyx mori* (silkworm)	77 (202/262)	5*e* − 51	Antioxidative stress tolerance	Selenocysteine biosynthesis	[29]

MRSW-530 (389 bp)	Ubiquitin-fold modifier 1 putative	*Callinectes sapidus *(blue crab)	81 (153/189)	7*e* − 30	Heat tolerance and aridity tolerance	ER stress-induced apoptosis	[30]

MRSW-582 (171 bp)	Cysteinyl-tRNA synthetase	*Danio rerio *(zebrafish)	72 (119/164)	5*e* − 14	Salt tolerance	tRNA aminoacylation	[31]

Bp: base pair: SSH: suppression subtractive hybridization.

**Table 3:** Identified transcripts showing significant homology with unannotated ESTs.

Clone number (length)	% identity (length)	*e*-value	Species	GenBank acc. no.	Tissue
MRSW-19 (168 bp)	79 (48 bp)	0.013	*Rhopalosiphum padi*	FO069008	Whole body
MRSW-54 (354 bp)	79 (168 bp)	2*e* − 43	*Litopenaeus vannamei*	FE070435	Eyestalk
MRSW-66 (300 bp)	67 (192 bp)	4*e* − 13	*Homarus americanus*	FE535181	Gill, epipodite, branchiostegite, heart, ovary, testis, antennal gland, abdominal muscle, hepatopancreas, and brain
MRSW-95 (458 bp)	99 (382 bp)	0.0	*Macrobrachium rosenbergii*	EL696133	Hemocyte
MRSW-198 (259 bp)	73 (130 bp)	1*e* − 18	*Penaeus monodon*	AW600771	Hemocytes
MRSW-285 (138 bp)	90 (117 bp)	1*e* − 40	*Macrobrachium nipponense*	FL589767	Ovary
MRSW-286 (354 bp)	70 (190 bp)	1*e* − 13	*Penaeus monodon*	EE663100	Lymphoid organ
MRSW-442 (177 bp)	77 (130 bp)	5*e* − 27	*Petrolisthes cinctipes*	FE785227	Pooled heart, gills, and whole crab
MRSW-558 (465 bp)	69 (234 bp)	3*e* − 29	*Carcinus maenas*	DN161325	Gill, hypodermis, heart, hepatopancreas, antennal gland, brain, testis, and muscle
MRSW-568 (105 bp)	84 (41 bp)	0.086	*Salmo salar*	EG810254	Thymus

bp: base pair.
